# Rehabilitation Oculomotor Screening Evaluation (ROSE)—A Proof-of-Principle Study for Acquired Brain Injuries

**DOI:** 10.3390/jcm13144254

**Published:** 2024-07-21

**Authors:** Tina Yu-Zhou Li, Kelsey Madge, Francesca Richard, Preeti Sarpal, Elizabeth Dannenbaum, Joyce Fung

**Affiliations:** 1 School of Physical and Occupational Therapy, McGill University, 3654 Prom Sir-William-Osler, Montréal, QC H3G 1Y5, Canada; tina.yz.li@mail.mcgill.ca (T.Y.-Z.L.); kelsey.madge@mail.mcgill.ca (K.M.); francesca.richard@mail.mcgill.ca (F.R.); preeti.sarpal@mail.mcgill.ca (P.S.); 2 Jewish Rehabilitation Hospital, Site of CISSS-Laval (Center of Integrated Health and Social Services of Laval) (CISSS-Laval), Research Site of the Montreal Center for Interdisciplinary Research in Rehabilitation (CRIR), Laval, QC H7V 1R2, Canada; edannenbaum_hjr@ssss.gouv.qc.ca

**Keywords:** oculomotor screening, acquired brain injury, stroke, traumatic brain injury, neurorehabilitation

## Abstract

**Background/Objectives:** Acquired brain injury (ABI) is a major cause of global disability. Many ABI patients exhibit oculomotor dysfunctions that impact their daily life and rehabilitation outcomes. Current clinical tools for oculomotor function (OMF) assessment are limited in their usability. In this proof-of-principle study, we aimed to develop an efficient tool for OMF screening and to assess the feasibility, acceptability, and relevance in a small sample of ABI and control participants. **Methods:** We created the Rehabilitation Oculomotor Screening Evaluation (ROSE) by reviewing existing OMF assessments. ROSE was pilot-tested on ABI patients (*n* = 10) and age-matched controls (*n* = 10). Data regarding the characteristics of the assessment, such as the duration, level of participant comprehension, and participant experience were also collected. **Results:** ROSE takes <20 min ($\bar{x}$ = 12.5), is easy to complete (agreement $\bar{x}$ = 4.6/5), and is well-accepted ($\bar{x}$ = 4.8/5). Patients scored higher in all subtests and total score ($\bar{x}$ = 34.8 for ABI vs. 8.9 for controls). Most subtests did not provoke any symptoms, especially for controls. There were no significant between-group differences in symptom provocation. This proof-of-principle study shows that ROSE is feasible, acceptable, and relevant for adult ABI patients. **Conclusions:** ROSE needs further evaluation for reliability testing and validation in larger samples and diverse neurological conditions. Establishing norms for various ages, sexes, and populations should be considered for the deployment of ROSE as an OMF clinical tool.

## 1. Introduction

The human oculomotor system involves interconnected regions throughout the central nervous system (CNS) that interact to control eye movements to be able to perceive the world [1,2]. Rather than simply responding to a stimulus, proactive eye movement strategies are used effectively to know where to look, what to look for, and what information is required for a particular action [3,4]. Based on current literature, eye movement strategies consist mainly of smooth pursuits, saccades, vergence (convergence and divergence), and the vestibulo–ocular reflex (VOR) [3]; for more details, see Table 1.

Acquired brain injury (ABI) implicates damage to the brain that has occurred after birth and consists of both non-traumatic and traumatic brain injury (TBI), as well as cerebrovascular accident (CVA) [6]. ABI is recognized as one of the leading causes of death and disability for Canadians under 40 years old, with an incidence of approximately 165,000 people per year [7]. Depending on the nature of a brain injury, various areas and associated functions may be adversely affected [6,8], often involving the oculomotor system. Patients with CVA and TBI are often referred to vestibular rehabilitation [8], which can be attributed to the high prevalence of oculomotor dysfunctions (OMDs), as seen in approximately 90% of ABI [8,9,10]. OMDs may manifest as nystagmus, strabismus, cranial nerve palsies, blindness, visual field loss, diplopia, headaches, dizziness, and visual perception deficits, among others [1,6,9]. In addition, OMDs can also adversely affect the performance of eye movements [9]. Consequently, individuals may experience increased difficulty completing their everyday activities and community participation, adversely impacting their quality of life [8]. It is important to recognize that OMD may play a pivotal role in functional vision and rehabilitation outcomes, especially in the ABI population. The early detection and intervention of OMD can markedly impact the patient’s recovery and overall quality of life [8]. The use of an OMD screening tool that is simple and easy to use clinically is needed for rehabilitation and to track progress.

Existing measures that can be used to assess OMD, such as the Oculomotor Assessment Tool (OMAT), Vestibular Oculomotor Screening (VOMS), Craig Hospital Eye Evaluation Rating Scale (CHEERS), and Ocular Motor Score (OMS), have been demonstrated to have a strong inter- and intra-rater agreement as well as test–retest reliability. However, there are many limitations that hinder their utility as a clinical assessment. For instance, OMAT measures saccades and pursuit movements while a consistent target point placement is needed to improve testing stability and outcome accuracy [2]. The OMAT is not only expensive to administer but also limited in evaluating only saccades and pursuits which are not the full spectrum of OMD. In contrast, VOMS is a valuable and quick on-field evaluation of OMD following sports-related concussion injuries. Despite the efficient assessment of eye movements within a brief 5-min window, the evaluation method relies predominantly on subjective judgment through symptom rating [11]. As such, this evaluation not only fails to address subtle OMD that can significantly impact function without causing pronounced symptoms but also fails to align with the latest measurement recommendations. Comparatively, the OMS serves as a structured clinical protocol tailored for assessing ocular motor functions in both children and young adults, encompassing a diverse array of functions [12]. More importantly, it integrates static features that include head position, eyelid function, pupillary responses, and the presence of strabismus [12]. OMS also introduces a graded scoring system, distinguishing it from alternative assessment tools. Nevertheless, the challenge of accessing the instruction protocol and the inherent ambiguity and lack of granularity in the ratings potentially hinder its ability to deliver comprehensive and informative outcomes. Similarly, CHEERS also uses objective numeric scoring to evaluate eye movement based on impairment degree [9]. What sets CHEERS apart is its approach of including specific details that include evaluating deficits in all movement directions for a thorough analysis instead of overall movement. For instance, it dissects smooth pursuits into elements like intrusion amplitude, intrusion count, and nystagmus [9]. This detailed breakdown provides better insights into oculomotor function and helps pinpoint areas of impairment. However, the clinical utility of CHEERS is questionable because it is time-consuming and the rating scale is not user-friendly.

Due to these limitations, the existing oculomotor function assessment tools, when considered individually, exhibit limitations that hinder their effectiveness in offering a comprehensive evaluation of OMF. This underscores the need to create an assessment tool that not only addresses these limitations but also advances the evaluation and treatment of individuals impacted by OMD. Thus, our primary objective of this study is to develop an assessment tool, Rehabilitation Oculomotor Screening Evaluation (ROSE) that surpasses the deficiencies inherent in existing tools, thereby fostering a more holistic approach to evaluating and addressing individuals with OMD within the realm of clinical practice. We intend to pilot and evaluate the new tool in terms of its feasibility, relevance, and applicability in ABI. Once ROSE is deemed feasible and acceptable as a screening tool, future studies will be conducted to refine the tool and test its reliability and validity in different neurological populations.

## 2. Materials and Methods

### 2.1. Literature Review

The initial phase of our project involved conducting an extensive search for scholarly articles, research papers, and studies related to oculomotor function assessments (Supplementary Materials File S1). We systematically compared the assessments based on six critical dimensions that aligned with our study objectives: the areas of assessment, scoring methods, target population, required equipment, and the psychometric properties supporting clinical applicability. This analysis allowed us to identify strengths and weaknesses for each evaluation and determine which aspects of assessment to incorporate into the development of our novel OMF assessment tool. From the analysis, ROSE emerged by enhancing and adapting the test components from the following four assessment tools: CHEERS, OMS, OMAT, and VOMS. The test components were revised to better suit our study objectives, integrate the latest recommendations from current literature, address any identified limitations, and incorporate over 20 years of clinical expertise from the two supervising physiotherapists.

### 2.2. Development of the ROSE Tool

The ROSE tool (Supplementary Materials File S2) was developed to measure OMDs commonly seen in ABI. The tool included 8 subtests grouped into 4 categories: (i) smooth pursuits + vergence (SPV), (ii) saccades (SC), (iii) cover tests (eye cover test and alternate cover uncover test) + gaze fixation (CTGF), and iv) VOR + cVOR (VORs). Numerical scores were assigned to the 8 subtests based on the degree of deviation from asymptomatic performance, with scores increasing as the severity worsened. Depending on the nature of subtests, the following components were measured and quantified on a scale of 0–2: movement quality (symmetry, fluidity, and accuracy), speed, reflexive movements, and distance. Moreover, any abnormalities detected (e.g., nystagmus, saccadic intrusions) were measured, and the presence of red flags and/or a score of 2 indicated the need for the patient to seek additional medical attention. A baseline/observation section was also included which noted static OMF domains. The subtotals for each of the 4 categories were summed into a total score, ranging from 0 (no deficits) to 48 (maximum deficits). Furthermore, we have introduced an adjusted total score of 44 (as opposed to the standard 48) for scenarios in which vergence testing is not feasible, as seen in conditions such as lazy eye, glass eye, or severe ptosis. This refinement contributes to a more comprehensive and normalized evaluation approach, accommodating a broader spectrum of clinical situations.

To be clinically relevant and practical, we chose tests requiring minimal and common office equipment: pen, printed “E” on paper or flashcard, flashlight, tape measure, chair, 30 cm ruler, timer (or app), metronome (or app), and an eye-shield (optional). Incorporating the principles that underscore the effectiveness of OMAT’s measurement tool in refining accuracy and standardization for saccades and vergences, we devised an alternative approach with a heightened focus on cost efficiency. This entailed the attachment of two small circular stickers to the ends of a standard 30 cm ruler. Furthermore, to align with the latest recommendations for the VOR test, we introduced the practice of having the examiner print a capital letter “E” in a 12-size Calibri font. This modification seeks to enhance the overall methodology while ensuring practicality and cost-effectiveness.

Prior to subject testing, the ROSE tool underwent a trial phase involving 20 healthy individuals, including family members, friends, hospital staff, and the research team. As there are no normative values in the literature for certain tests, the aim of this trial was to determine conservative estimates for quantifying the severity, such as the number of saccadic cycles within an 8 s interval. This contrasts with existing tools that solely rely on the subjective qualification of “observably slow” without reference to a defined normal speed. This trial testing enabled important adjustments and modifications to optimize the scoring of ROSE.

### 2.3. Participants

Ten participants, diagnosed with an ABI, were recruited from the traumatology and post-stroke rehabilitation programs at the Jewish Rehabilitation Hospital (JRH), a McGill-University-affiliated rehabilitation hospital in Laval, Quebec. They were matched with ten healthy controls by age and biological sex. Inclusion criteria for the ABI group were as follows: (i) medical diagnosis of an ABI (TBI or CVA); (ii) age between 18 and 80; and (iii) corrected vision of 20/40 or better. Exclusion criteria included: (i) severe ocular diseases that can impact vision or ocular motor performance (i.e., glaucoma, diabetic retinopathy, macular degeneration, etc.); (ii) inability to comprehend or speak English and/or French; (iii) inability to actively participate and follow instructions. Participants with specific vestibular diagnoses were not excluded from the study, but the diagnosis would have been noted down. Eligibility and exclusion criteria for the controls were identical to the ABI group, except for having a medical diagnosis of an ABI. Ethics approval was granted by the CRIR ethics review board. Written informed consent was obtained from all participants.

### 2.4. Administration and Assessment of the ROSE Tool

Each participant took part in one 30-min assessment session at the JRH that followed the established administration guidelines. During the administration process, five senior students in physical or occupational therapy were supervised by a physiotherapist (specialized in vestibular rehabilitation) in case of adverse events needing immediate professional intervention. Participant demographic information, including age, biological sex, diagnosis, glasses worn, visual acuity score, and pertinent medical history, was gathered before the evaluation. Subsequently, the ROSE tool was administered to each participant by a pair of students with one conducting the assessment and another observing the process. This observer meticulously documented pertinent assessment metrics and observations, encompassing the overall assessment duration, frequency of patient questions, instances of examiner test repetition or additional clarification, and the duration and intensity of symptom provocation. Test execution was contingent on patient availability and the resources accessible within the hospital. It is noteworthy that the test environment took place in a clinical setting that was difficult to enforce homogeneity, encompassing a blend of enclosed and open settings, and spanning various times of the day. Additionally, specific assessment instances coincided with the simultaneous activities of therapists. However, that was realistic in most clinical environments.

During the testing process, after completing each of the four categories, participants were directed to complete a visual analog scale (VAS) of their provoked symptoms. Participants placed a vertical mark on a 10-cm line to denote the perceived intensity of symptoms (from none/minimal to maximal) they encountered in each test category. The measurement was taken from the zero endpoint to the marked point and rounded to the nearest 0.5 mm. Participants were additionally asked about which test(s) within the categories provoked the most response. To take into consideration adverse events that can impact assessment performance, we incorporated a baseline VAS evaluating any perceived symptom provocation within the last 24 h. We opted for the utilization of a VAS over the numeric rating scale (NRS) utilized in VOMS due to its inherent simplicity and intuitive design. The heightened sensitivity in VAS allows clinicians to discern even subtle shifts in patients’ perceptions or experiences [13]. The total scores and the VAS scores for all participants were derived by summing the scores of the four subtest categories. After the assessment, the participants were asked to rate their responses to a questionnaire regarding their experience (Table 2) using a Likert scale. Assessment data and feedback responses were anonymized and digitalized to allow easy access during the analysis phase.

### 2.5. Data Analysis

Client acceptance was evaluated by comparing the number of approached eligible participants to the number of eligible participants who participated in the study. If more than 50% of approached eligible participants refuse to move forward to the consent process and/or participate in the assessment trial, the assessment tool would be deemed to have low client acceptance.

We also evaluated item clarity based on the information/data gathered by the second student who observed the assessment administration. If more than 20% of the total participants found a specific item difficult to understand or required 3 or more repetitions of the instructions, the specific item will be denoted as “unclear” or “difficult to understand”. The concept of clarity was analyzed as complicated instructions could serve as a barrier to the efficiency and ease of administration, especially for a population with ABI. Next, timeliness was determined based on the mean duration from the total administration periods. The assessment was deemed “too long” if the mean total administration duration was greater than 20 min.

Furthermore, the symptom provocation data collected from the ROSE tool administration was evaluated. The ROSE tool measured symptom provocation for groups of subtests (SPV, SC, CTGF, VORs). In the tool, the participants are asked if specific symptoms were elicited by the subtest groups, and to rate the intensity of the symptom by marking a vertical line on a visual analog scale. Distances on the visual analog scale measuring the intensity of the symptom provocation were calculated and compared among participant groups (individuals with ABI and controls). Non-parametric tests were carried out in this small sample to detect potential differences between controls and ABI patients. Based on the results of this pilot study, the effect size will be estimated, and a power-based sample size calculation will be carried out for a future validation study. Information on symptom provocation would also indicate whether appropriate assessment items were included to create a feasible test. If multiple assessment items demonstrate high provocation of symptoms, it can likely also influence both the acceptability and length of the assessment. Secondly, the order of items in the assessments in comparison to the symptom provocation will also be analyzed to evaluate if the order of the items could be influencing symptom provocation. This information could inform if the order of the items in the ROSE tool needs to be adjusted.

The post-assessment questionnaire (Table 2) administered at the end of the sessions provided information about the general feelings of the participants towards the assessment. The responses would be scored using a Likert scale and calculations were carried out to determine the clarity, symptom provocation, administration time, and acceptability of the test from the participants’ perspectives.

## 3. Results

### 3.1. Participant Characteristics

**Figure 1:**
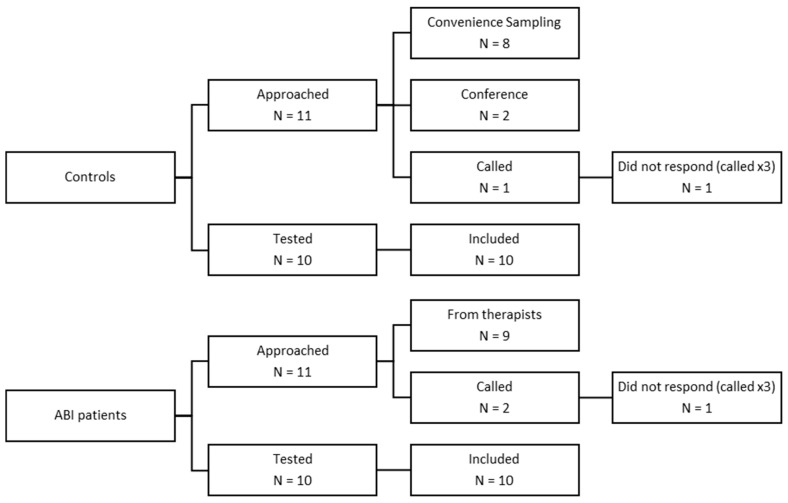
Recruitment and participation in the controls and ABI groups.

Figure 1 illustrates the recruitment process, including the number of participants approached, tested, and included in each group.

The ABI group included 10 participants, with a mean age of 49.6 (±17.39) years old. Six of them were female. Of the 10 patients, six were diagnosed with CVA and four others with TBI. Four of the patients (40%) presented with some sort of double vision and seven (70%) wore glasses. The control group was matched with the ABI group by mean age (47.2 ± 13.78) and sex distribution (χ^2^ = 0.278) (Table 3).

### 3.2. ROSE Scores

Since our samples were small and without normal distributions, we performed a Mann–Whitney U test to test our hypotheses. The scores were all converted into percentages as they did not all have the same denominator (for patients who could not complete parts of ROSE).

**Figure 2:**
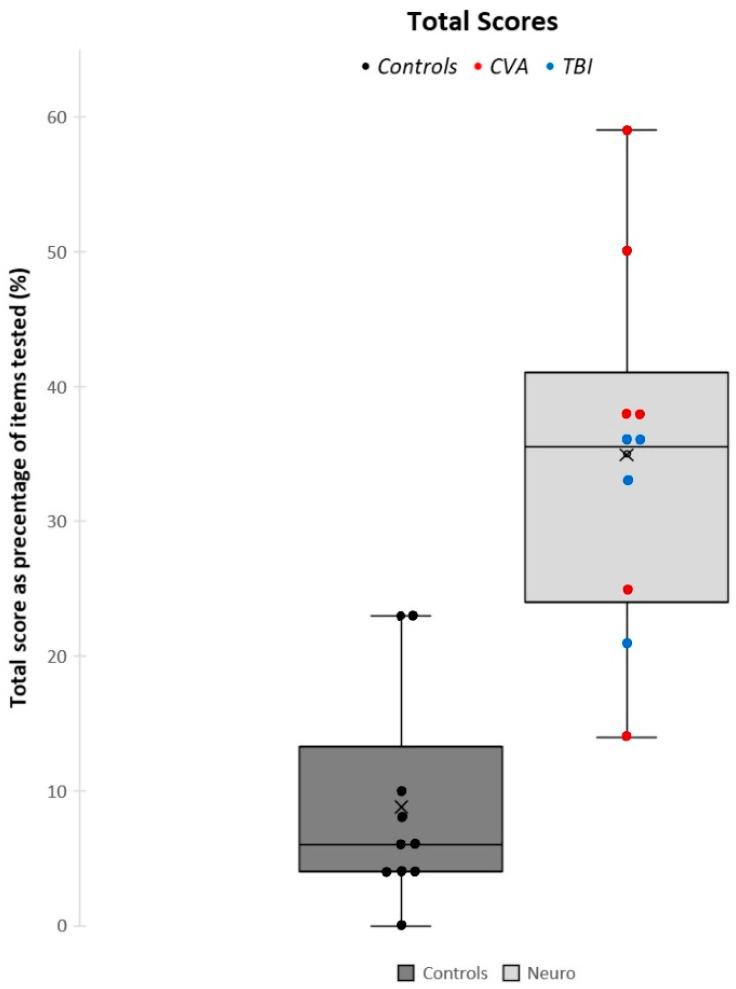
Comparison between the mean scores of ROSE total scores for both the control and ABI groups.

**Figure 3:**
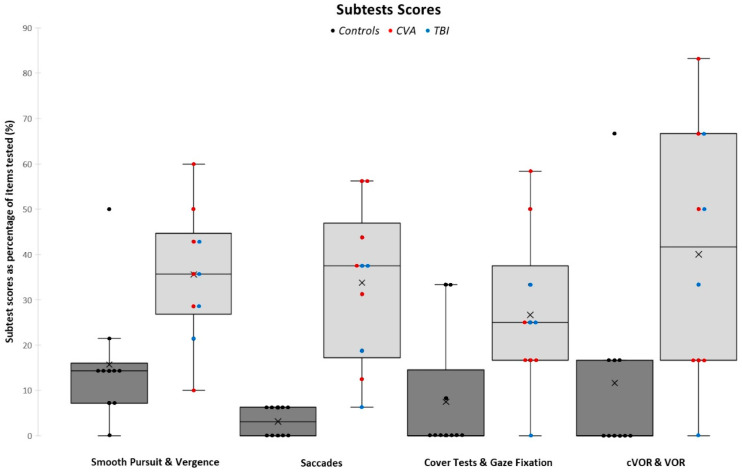
Comparison between the mean scores of ROSE subtest categorical scores for both the control and ABI groups.

The Mann–Whitney U test showed a significant difference (*p* < 0.001) between the mean ROSE total scores of the ABI group (34.80%) and the control group (8.96%), as depicted in Figure 2. There was also a significant difference between the two groups for all four subtest categories: SPV (*p* = 0.007), SC (*p* < 0.001), CTGF (*p* = 0.019), and VORs (*p* = 0.015), as shown in Figure 3.

### 3.3. ROSE Symptom Provocation

**Figure 4:**
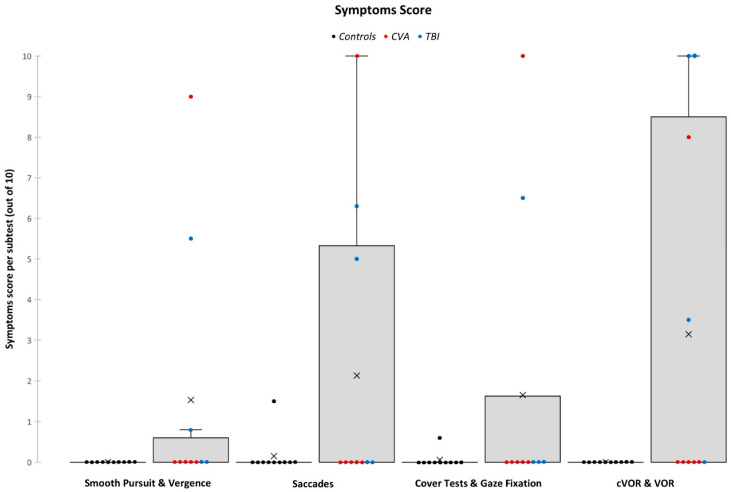
Comparison between the mean scores of ROSE subtest categorical symptom scores for both the controls and ABI patients group.

In terms of symptom provocation, the scores were also converted to percentages. As the total score includes a baseline symptom score, only the subtest categorical scores were included in the analysis to provide a comprehensive view of how ROSE provoked symptoms in the two groups. The Mann–Whitney U test showed that there were no significant differences in any of the four subtest categories (Figure 4): SPV (*p* = 0.280), SC (*p* = 0.393), CTGF (*p* = 0.684), and VORs (*p* = 0.143). Even so, the ABI group scored overall higher than the control group, indicating a potential clinical significance.

In terms of frequency of provocation, the information was compiled from the data sheets, and Table 4 illustrates that saccades and cVOR were the two test components that provoked symptoms most frequently. However, none of the test components provoked symptoms in more than 3 of the 20 trials.

### 3.4. ROSE Clarity of Items

Table 5 illustrates the clarity of different ROSE subtests in terms of the amount of instruction repetition needed. The repetitions were divided into three main reasons: for clarity purposes, due to patients’ miscomprehension, and for examiner verification purposes. Most task instructions were clear, without the need for repetitions in more than two trials in terms of clarity and miscomprehension issues. There was also a similar number of repetitions in the controls and ABI groups for these two reasons. However, in terms of examiner verification, there were six repetitions in the ABI group and none in the control group.

### 3.5. ROSE Administration Time

Table 6 shows the time of administration for both the control group and ABI group, and overall. The targeted ideal administration time was below 20 min. In the control group, this was met with a mean of 8:52 (ranging from 6:56 to 12:04). In the ABI group, although the average of 16:05 (ranging from 10:18 to 27:47) was below the 20 min cutoff, 3 out of the 10 trials went over 20 min. However, among the three trials that surpassed the time limit, each included extended breaks: two of these involved patients with severe symptoms, and the third involved a patient experiencing eye fatigue.

### 3.6. ROSE Post-Assessment Questionnaire

The post-assessment Likert-scale responses were quantified from 1 to 5, with 5 representing strongly agree and 1 representing strongly disagree.

Most (19/20) participants strongly agreed (*n* = 14) or agreed (*n* = 5) that the assessment was easy to complete ($\bar{x}$ = 4.6/5), and one participant disagreed; however, they attributed the difficulty to the increased symptom provocation rather than a lack of understanding. All participants strongly agreed or agreed that the instructions were easy to understand and follow ($\bar{x}$ = 4.85/5), the assessment was completed within a reasonable time frame ($\bar{x}$ = 4.85/5), and that they would be willing to be assessed with the tool again ($\bar{x}$ = 4.8/5).

**Figure 5:**
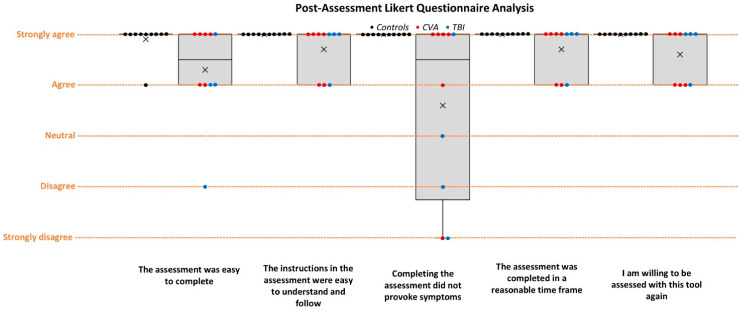
Comparison of post-assessment Likert questionnaire agreement between control and ABI groups.

In terms of symptom provocation, all controls strongly agreed that the assessment did not provoke symptoms ($\bar{x}$ = 5/5). Two of the ABI participants strongly disagreed, one disagreed, and one was neutral ($\bar{x}$ = 3.6/5). The lowest two scores both experienced severe symptoms: one being provoked by a busy and noisy environment and the other from being symptomatic post physiotherapy session prior to the assessment. The other two patients who did not agree also experienced symptoms (Figure 5).

The controls scored between 4.9 and 5 for all five questions. On the other hand, the patients’ scores ranged between 3.6 and 4.7. Overall, all the questions obtained a score over 4, ranging from 4.3 to 4.85 (Table 7).

## 4. Discussion

The comparison of ROSE scores between individuals with ABI and the control group demonstrates ROSE’s ability to distinguish between normal and abnormal oculomotor function, even within the limitation of small sample sizes. While reliability testing of ROSE is not an objective of this proof-of-principle study, the subtests that comprised the total score were extracted and refined from existing tools with strong inter- and intra-reliability. Nevertheless, to establish the validity and reliability of ROSE as a clinical tool, further investigation in a larger sample of TBI patients with different severity is underway. Additionally, the absence of a significant difference in symptom provocation between the ABI and control groups, along with infrequent symptom elicitation, highlights the low symptom-provoking nature of the ROSE assessment. This characteristic is important and clinically relevant, as existing tools with their limitations of symptom provocation and measurement complexity hinder acceptance both among clients and within the clinical community. Consequently, the minimal symptom provocation associated with the ROSE assessment enhances the tool’s feasibility and acceptability. Moreover, this underscores the remarkable efficacy of the ROSE assessment in detecting OMD without inducing discomfort in subjects. This contrasts with other tools such as VOMS, which primarily assess dysfunction by provoking symptoms. ROSE enables clinicians to effectively monitor OMD and address the impact of dysfunction on functionality, all without subjecting individuals to the discomfort that could otherwise compromise their perception of quality-of-life post-treatment. This distinction showcases the patient-centered focus of the ROSE assessment, highlighting its potential to enhance both diagnostic accuracy and patient comfort in clinical practice.

Despite the generally modest level of symptom provocation, it is noteworthy that the subtests eliciting the most frequent symptoms were saccades, cVOR, and smooth pursuits. This observation raises the possibility of resequencing the subtests, placing these three at the conclusion of the assessment, or modifying the tests to mitigate intensity. Nevertheless, it is important to acknowledge that the frequency of symptom provocation remained below 3/10. As such, this frequency does not raise concerns regarding an immediate procedure change. It is also important to acknowledge that instances of symptom provocation observed during the administration of ROSE were occasionally attributed to the presence of noise and activity within the testing environment. This observation demonstrates the importance of a quiet and calm testing environment. As a result, the merits of standardizing the testing environment to ensure consistent and reliable outcomes should be considered.

In terms of clarity, the findings derived from both the post-assessment questionnaire and the data sheet demonstrate the ease of comprehension and execution of ROSE, as perceived by the participants. However, it is noteworthy that repeating instructions to avoid head movement could prove advantageous when working with this specific population. Nevertheless, the findings do uncover that a predominant share of challenges encountered during the assessment can be attributed to the examiner, as shown by the frequency of subtest repetitions stemming from examiner-related factors. This can be attributed to the inherent complexity of identifying subtle and rapid eye abnormalities. The need for repeated attempts due to examiner-related factors was particularly evident during the cVOR and VOR subtests. This can be linked to the heightened demands placed on examiners during these test protocols, as the examiner must simultaneously monitor oculomotor function while guiding and coordinating participants’ movements. Furthermore, some ABI patients had difficulties performing the cVOR-instructed movements due to CVA-related conditions, such as hemiparesis, spasticity, or flaccidity. As such, an alternative approach for the evaluation of the cVOR was implemented, focusing solely on neck/head movements to facilitate task completion. To address this, in future iterations, the assessment instructions should incorporate an alternative version of the cVOR assessment, along with a heightened emphasis on including increased reminders for specific instructions.

Regarding administration time, although a few surpassed our target, overall, ROSE attained our goal of being administered within 20 min. This suggests that ROSE is an efficient and simple tool to use in the rehabilitation setting. Furthermore, from the participants’ high adherence to the study and agreement seen in the post-assessment questionnaire, ROSE demonstrates high client acceptability.

Finally, in terms of ROSE as a useful test in the clinical rehabilitation setting, many comments and suggestions were received from fellow therapists after presenting the ROSE in subsequent knowledge dissemination workshops. Many pointed out the lack of a section evaluating neglect. Hence, after considering the different comments received, we have added a new section evaluating neglect in eight gaze directions, bringing up the total score of the test to 50 (Supplementary Materials File S3). This modified version of ROSE would be able to assess OMD in more detail.

The creation of the ROSE is an important step in the current literature as it stands as one of the only standardized tests for the OMS and can assess both the quantitative (scoring movement quality and abnormalities) and qualitative (ratings of symptoms provocation) aspects of the OMS. The ROSE can be used by all professionals who need a tool to assess the OMS of patients and can be used to evaluate and track progress throughout rehabilitation processes.

### Limitations

Further validation of ROSE within the neurological population remains imperative. This necessitates validation with a larger sample size and ABI severity, given the current study’s limited convenient sample of only 10 patients. Additionally, extending the assessment to encompass diverse neurological conditions beyond stroke and TBI is vital for comprehensive validation. Furthermore, the newest modified version of the ROSE (Supplementary Materials File S3) has not been piloted nor validated and would also need more extensive testing in the future.

In light of ROSE’s progression into the subsequent phase, certain crucial considerations come to the fore. Firstly, careful attention must be given to potential time constraints, including participant recruitment and meticulous analysis. Adequate time allocation ensures robustness in research outcomes. Secondly, while the pragmatic approach of grouping participants within a single setting is advantageous, it’s imperative to minimize any potential impact on results, considering the pilot study nature of this research. Furthermore, the lack of standardized testing environments, as highlighted earlier, could potentially influence symptom provocation outcomes. This variable environment might also impact the timing of administration and overall acceptability of ROSE. Cardiovascular risk factors should also have been noted in both the control group and the ABI group to ensure that no disparities in results could have been a result of those factors.

From this pilot test, it’s evident that ROSE is feasible, acceptable, and relevant. Moreover, it has demonstrated the ability to preliminarily discern a significant scoring distinction between the control and ABI groups. However, due to the limitations of our small sample size, a comprehensive evaluation of differences between the CVA and TBI populations was not feasible within this study. Nonetheless, exploring these differences could hold valuable implications for future research directions. Current recommendations for best practices [14] should also be taken into consideration before generalizing ROSE as an OMD screening tool in neurorehabilitation.

## 5. Conclusions

From this pilot test, we can see that the ROSE test is a feasible, acceptable, and relevant test. It was also able to preliminarily determine a significant difference in terms of scoring between the control and ABI groups. In the future, the validity and reliability of ROSE should be further assessed. Establishing normative cutoff scores for both total and subtest of ROSE scores, serving as a quantifiable and meaningful metric, could also be a step to take.

Despite its limitations, the evolution of ROSE is ongoing as we conduct a larger study with the scale in TBI adults. This significant advancement underscores the active transition of this assessment toward practical real-world application.

## Figures and Tables

**Table 1:** Eye movements defined [3,5].

Eye Movements	Definition
Smooth pursuit	Tracking and following a moving object with our eyes in a smooth, coordinated manner. An example of smooth pursuits used in function includes following the movement of the ball during a soccer game.
Saccades	Ballistic eye movements that abruptly change from one point of interest to another. Examples of saccades in function include reading, driving, and navigating in a busy environment.
Convergence and Divergence	The coordinated inward or outward movement of both eyes to maintain gaze on an object moving towards or away from you (depth perception). Examples of vergence in function include participating in activities including catch, tennis, and volleyball.
Vestibulo-ocular reflex (VOR)	The VOR helps maintain a stable visual field during head movements by allowing the eyes to move in the opposite direction of the movement. For example, reading a street sign while driving or looking at your surroundings while running.
Cancellation of VOR (cVOR)	The cVOR is a process that allows the gaze to remain oriented with head movements. For example, looking both ways before crossing the road or when turning our head to look at something.

**Table 2:** Post-assessment questionnaire.

	Strongly Agree	Agree	Neutral	Disagree	Strongly Disagree	Comments:
The assessment was easy to complete.						
The instructions in the assessment were easy to understand and follow.						
Completing the assessment did not provoke symptoms.						
The assessment was completed in a reasonable time frame.						
I am willing to be assessed with this tool again.						
I perceive this tool to be a useful assessment in rehabilitation.						

**Table 3:** Sociodemographic characteristics of participants.

		Statistics	Controls	ABI	Overall	*p*-Value	Chi-Square
Age (Years)		N	10	10	20	1.000	
		Mean	47.2	49.6	48.4		
		Std. Deviation	13.78244	17.39221	15.3225		
		Minimum	28	20	20		
		Maximum	71	80	80		
Sex							
	Male	n	4	6	10		0.278
	Female	n	6	4	10		
Diagnosis							
	Stroke	n	n/a	6			
	TBI	n	n/a	4			
Presence of Double Vision							
	Vertical	n		1			
	Horizontal	n		2			
	Both	n		1			
Relevant medical history and comorbidities							
	Cataracts	n		1	1		
	Cardiac S/S	n	1 (HTN)	1 (A-Fib)	2		
	Diabetes	n	1		1		
	Migraines	n	1	1	2		
	Autoimmune Condition	n	1		1		
Glasses			7	7			

**Table 4:** Frequency of symptom provocation by ROSE test components.

Test Components	Frequency
Smooth Pursuit	2
Vergence	1
Saccades	3
Gaze Fixation	1
Cover test	0
Alternate cover test	0
cVOR	3
VOR	1

**Table 5:** Comparison of task instruction repetition between the controls and ABI patients group according to three different reasons. (Legend: Controls in black, CVA in red, TBI in blue).

	Clarity		Miscomprehension		Examiner Verification	
ROSE Subtests	Controls (/10)	ABI (/10)	Controls (/10)	ABI (/10)	Controls (/10)	ABI (/10)
Smooth Pursuit						
Vergence						1
Saccades	1					
Gaze Fixation	1		1			
Cover Test						1
Alternate Cover Test						
cVOR		1		1		2
VOR						1 + 1

**Table 6:** Comparison of administration time between the control and ABI groups.

	Control (min: s)	ABI (min: s)	All (min: s)
n	10	10	20
Mean	8:52	16:05	12:29
Minimum	6:56	10:18	6:56
Maximum	12:04	27:47	27:47

**Table 7:** Post-assessment Likert questionnaire analysis between control and ABI groups.

Group		Q1-Post ^i^	Q2-Post ^ii^	Q3-Post ^iii^	Q4-Post ^iv^	Q5-Post ^v^
Control	Mean	4.9000	5.0000	5.0000	5.0000	5.0000
	N	10	10	10	10	10
	Std. Deviation	0.31623	0.00000	0.00000	0.00000	0.00000
	Minimum	4.00	5.00	5.00	5.00	5.00
	Maximum	5.00	5.00	5.00	5.00	5.00
ABI	Mean	4.3000	4.7000	3.6000	4.7000	4.6000
	N	10	10	10	10	10
	Std. Deviation	0.94868	0.48305	1.71270	0.48305	0.51640
	Minimum	2.00	4.00	1.00	4.00	4.00
	Maximum	5.00	5.00	5.00	5.00	5.00
Total	Mean	4.6000	4.8500	4.3000	4.8500	4.8000
	N	20	20	20	20	20
	Std. Deviation	0.75394	0.36635	1.38031	0.36635	0.41039
	Minimum	2.00	4.00	1.00	4.00	4.00
	Maximum	5.00	5.00	5.00	5.00	5.00

^i^ Q1: The assessment was easy to complete. ^ii^ Q2: The instructions in the assessment were easy to understand and follow. ^iii^ Q3: Completing the assessment did not provoke symptoms. ^iv^ Q4: The assessment was completed in a reasonable time frame.^v^ Q5: I am willing to be assessed with this tool again.

## Data Availability

No new data are collected. Anonymized and stored data could be retrieved from an internal database.

## Supplementary Materials

The following supporting information can be downloaded at: https://www.mdpi.com/article/10.3390/jcm13144254/s1. File S1: Oculomotor Summary Table. File S2: ROSE Tool. File S3: New ROSE Tool. File S4: General Scoring Sheet.
