# Supplementary material for: Rehabilitation Oculomotor Screening Evaluation (ROSE)—A Proof-of-Principle Study for Acquired Brain Injuries
Source: J Clin Med. 2024 Jul 21;13(14):4254. doi: 10.3390/jcm13144254 (PMC11278066; doi:10.3390/jcm13144254)
Supplement: Supplementary file 1 [file jcm-13-04254-s001.zip › File S1 - Oculomotor Summary Table.pdf]

Test	Subtests of Interest	Assessed Area	Scoring	Population Targeted + General Info	Admin Time	Equipment	Psychometric Properties	Pros	Cons	Notes
**OculoMotor Assessment Tool (OMAT)**	Saccadic eye movements, vergence jump	- NPC (Accommodative amplitude)<br>- Vergence jumps<br>- Saccadic eye movements	- Measured values<br>- Similar to VOMS, perform a test followed by self-reported rating of symptoms provoked (0-10)<br>- Comparing endurance measures in terms of the first and latter 30s	Patients with persistent concussive symptoms; can be used in patients without concussion	5 mins	- OMAT tool vergence bars (two long bars and a small and large slider)<br>- Timer<br>- OMAT app<br>- OMAT card	n/a	- Saccadic movements and vergence jumps have been studies with general healthy population to determine normative data ranges (see Tables)<br>- Testing for NPC more in line with current guidelines (vs VOMS)<br>- Utilizes consistent targets for performing horizontal/vertical saccades and vergence eye movements (specifically, vergence jumps	- Costly material<br>- Needs time to setup<br>- Scarce research on the validity and reliability of the tool	- OMAT developed to overcome limitations of VOMS<br>- Statistically significant differences between adults & children (in terms of saccades)
**Vestibular Ocular Motor Screening (VOMS)**	All 7 items, but add adjustments like ways to quantify everything else except NPC<br><br>- Subjective Questionnai re	- Baseline symptoms<br>- Horizontal & Vertical Smooth pursuit (SP)<br>- Horizontal & vertical saccades<br>- NPC<br>- VOR (vertical & horizontal)<br>- Visual motion sensitivity	Symptom provocation for headache, dizziness, nausea, fogginess on a 0-10 scale. Likert scales, with 0 meaning no symptoms at present.<br><br>Convergence score measured based on the average measurement of 3 trials of near-point convergence distance (centimeters)	Patients with persistence symptoms post-concus sion (sport related?)<br><br>Assessment of the vestibular and oculomotor systems in both adolescent and collegiate athlete population	5 mins	- Target w/ 14 point font print<br>- Tape measure (cm)<br>- Metrono me	- Strong internal consistency<br>- NPC has a high intraclass correlation coefficient of about 0.9 within and between sessions.<br>- Good reliability shown for NPC<br>- Minimum detectable change of 4 cm → confidence in the diagnostic utility of NPC distance in diagnosing convergence insufficiency needs to be supported by other findings (i.e., symptom provocation or observations)<br>- Internal consistency estimates are excellent across each item<br>- Test-retest reliability between moderate to good across each item	- Strong internal consistency and significant correlation with the Post-Concussion Symptom Scale<br>- Ability to potentially differentiate concussed patients from healthy controls<br>- Useful in tracking recovery<br>- Recording symptomatology is important for the care of patients, it is subjective and hence typically leads to more variability compared to objective measurements.	- Lack of any testing for accommodative function<br>- Non-standard protocol for assessment of NPC (inconsistent with current standard practice)<br>- The patient should not hold the target because this may lead to a smaller NPC measurement (due proximal vergence)<br>- Target size (14-point font) recommended for the VOMS is considerably larger than current recommendations.<br>- The subjective nature of a patient's response could lead to either false positives or false negatives<br>- A person with less severe symptoms may not evoke any symptoms after a brief test, leading to a false negative<br>- The visual targets can lead to inconsistencies between various examiners due to physical differences between examiners, and within a single examiner from session to session.<br>- Does not assess the dynamics of the vergence system	- The number of eye movements for all subtests (both saccade conditions and vergence jumps) were not dependent on a participant's sex.<br>- Age was not a factor for the saccadic eye movements but was identified as a significant covariate for vergence jumps
**VOMS-C**	As VOMS	- Baseline symptoms<br>- Smooth pursuits<br>- Saccades (vertical and horizontal)	Yes or No response to provocation of headache, dizziness or nausea for each domain.	Symptomati c concussion patients between 5-9 years old.	5 mins	- Target w/ 14 point font print<br>- Tape measure (cm)	- Good accuracy to distinguish between concussed patients from controls, having good predictability<br>- 20% false positive rate among health controls (consistent with findings for adolescents)	- Minimal equipment needed (low cost)<br>- Can adequately distinguish between concussed and non-concussed patients<br>- Good predictability	- Significant false positive rate for more than 1 item<br>- Challenging to perform in this subset of patients (i.e., young children)<br>- No predictive risk factors have	- Involves collecting information regarding family depression history, migraine history, family migraine history and anxiety history<br>- Only family anxiety

		- Convergence<br>- VOR (vertical and horizontal)<br>- Visual motion sensitive				- Metronome		- Clinically useful adaptation of VOMS for children between 5-9yrs	been identified as of yet<br>- Fairly new adaptation, therefore limited information regarding psychometrics	history was seen to be related to high false positives
**King-Devick Test**	n/a	- Attention/Language through rapid number naming<br>- Saccadic eye movements	Participants are asked to read the numbers aloud quickly and accurately.<br><br>All three test card scores are summed for a total score. The total time to complete each card is recorded + the number of reading errors<br><br>The time and number of errors of words readare compared with either age-matched norms or baseline performance	Is a baseline measure and concussion sideline screening assessment test in pediatrics 5-17 y.o., adults 18+ yrs	2 mins	- Stop watch<br>- Score cards<br>- 4 cards: 1 practice card followed by 3 test cards<br>- Each card has 8 rows of 5 single-digit numbers	- Reliable test with modest learning effect over time<br>- Good convergent and discriminant validity in a diagnostically heterogeneous outpatient sample<br><br>Concurrent Validity<br>- Healthy controls (Rizzo,2016; n=42; mean age=32 (range 18-53)years)<br>- Excellent correlation with inter saccadic intervals<br>- Excellent predictive validity of K-D Test times with combination of inter saccadic intervals and number of saccades<br><br>Excellent test-retest reliability<br>- Healthy Adolescent (ICC=.81)<br>- Healthy Individuals (14-24 yrs) (ICC=.95)<br>- High School Athletes (ICC=.89)<br>- Adolescent Athletes (ICC=.92)	- Brief & valid measure of saccadic eye movements<br>- Has strong associations with Post-Concussion<br>- An objective measure of timed saccadic eye movements after SRC<br>- Test does not include an assessment of symptom provocation	- Do not measure pursuit, convergence, accommodation (limited in screening capacity)<br>- Highly susceptible to practice effect<br>- Does not include assessment of symptom provocation<br>- Limited as a measure of only 1 component of oculomotor impairment and does not include any vestibular components<br>- Do not measure oculomotor function, such as pursuits, convergence, and accommodation, and therefore may be limited in their screening capability<br>- Highly susceptible to practice effects, noting performance improvement with subsequent test administrations.	- Measures anticipatory saccades via scanning and rapid number naming over 3 trials with progressively increasing reading difficulty due to pattern variability<br>- Assess isolated saccadic eye movements under fixed test conditions
**Ocular Motor Score (OMS)**	#6, 7, 8, 9, 10, 11, 13, 14<br><br>#12? (need prism)	<u>Tasks (5 static + 10 dynamic)</u>:<br>1. Head posture<br>2. Eyelid position<br>3. Stereo visual acuity<br>4. Pupil response<br>5. Strabismus<br>6. Ocular motility range<br>7. Fixation in primary position (pp)<br>8. Fixation in 8 gaze directions<br>9. Saccades<br>10. SP<br>11. Convergence<br>12. Fusion<br>13. VOR<br>14. Cancellation of VOR<br>15. Optokinetic nystagmus (OKN)	Each scored 0 (normal), 0.3/0.5 (subnormal) or 1 (pathological) as estimated by visual inspection<br><br>Total (tOMS): 15 (healthy teenager/adult is believed to show a score of 0 or close to it)	Evaluates ocular motor function (for general population and population with neuropediatric disorders, young adult and child)<br><br>screening and follow-up of children with or suspected neuro pediatric disorders	~ 15-20 mins	- Lang Stereo Test<br>- Prism<br>- optokinetic drum or stripe<br>- A chair that can be rotated (CVOR)<br>- Pen lamp, cover	- Substantial to high overall observed intrarater (87%) and interrater (80%) agreement<br>- Subtests with higher overall disagreement: saccades, SP, head posture, fixation in 8 gazes<br>- Subtests with higher interrater disagreement: convergence, fusion test, VOR<br>- Most disagreement lies deciding between normal or subnormal	- Subtests are easy to perform<br>- Targets multiple aspects of oculomotor function (in dynamic portion)<br>- Used for people with neurological conditions (although have age-related factor, see cons)	- Requires extra tools such as the Lang stereo test (expensive) and optokinetic drum/stripe<br>- For children and young adults, not sure about validity for older adults<br>- Does not assess baseline symptom intensity	- There are age-related differences in tOMS (as the maturation of ocular motor system parallels with the development of the fovea)<br>- Most significant differences (for population with neuro pediatric disorders) seen in the dynamic subtests<br>- Pupillary light response & prism fusion test were sometimes difficult to evaluate due to reflections from recordings, results often excluded from evaluation (should the test be done in clinic, would likely not have the issue)<br>- However, video recordings are very helpful to assess for ocular motor function
**Craig Hospital Eye**	Highly based on all	- SP<br>- Amplitude of	Direction of movement, laterality	Measure eye	Within 5-7	Tape measure	- Very strong inter rater agreement<br>- Strong positive monotonic	- Assess several different oculomotor functions	- Not validated by readirect in person assessment (tested using	- Smaller sample size<br>- Very similar to our targeted

**Evaluation Rating Scale (CHEERS)**	Add symptoms provoked<br>Try to make more objective certain quantitative data (such as saccades speed)	saccadic intrusion(SI)<br>- Number of SI<br>- Gaze evoked nystagmus<br>- ROM<br>- Saccades<br>- Speed<br>- Amplitude<br>- Convergence<br>- VOR<br>- Fixation<br>- Constancy	(R vs L), speed assigned for each motility type.<br><br>Subtest scores: Rated on scales from 0-3 that are weighted proportionally to severity of deficit.<br><br>Final scores: 0 (no deficit) to 48 (max deficit in all scale)	movement abnormalities frequently seen in TBI<br><br>Neuro-ophthalmic deficits in acute TBI<br><br>(tested on population of 18-65 yo)	mins	(inches)<br>Metronome<br><br>visual acuity letter target (20/40)<br><br>2 small objects (undefined)	correlation between test-retest ratings<br>- 100% agreement interrater on presence/absence of ocular motility dysfunction<br>- Significant interrater agreement for any ocular motility abnormality identified	- Has a way to quantitatively describe movements (in terms of amplitude, number evoked, ROM, speed, time)<br>- Designed with expert input from specialists in neuro-optometry, neuro ophthalmology, occupational therapy, and physical rehabilitation medicine	video recordings)<br>- *the scale's validity precludes generalizability of conclusions to a larger TBI population*<br>- Some uncertainty in instructions in terms of mono vs binocular<br>- Some nystagmus & strabismus wen't underdetected due to examiner only evaluate the other eye	population and to our study objectives & design
**The North-Eastern Stage University College of Optometry Oculomotor Test**		-Ability<br>-Accuracy<br>-Head Movement<br>-Body Movements				The targets used during testing, known as Wolff wands consists of 1/2 cm. gold and silver spheres attached to dowel rods.		not being cognitively loaded by a knowledge of written symbols	subjective	
**OMA (current JRH tool)**		- Eye movements<br>- SP<br>- Saccades<br>- OKN<br>- Convergence-divergence<br>- Cover-uncover<br>- Alternating cover<br>- Near point fixation<br>- Head thrust<br>- Dynamic visual acuity<br>- VMS/VORc						- Evaluates many different oculomotor functions<br>- Evaluates quality and symptom reproduction	- Not really quantitative information	
