# Supplementary material for: Rehabilitation Oculomotor Screening Evaluation (ROSE)—A Proof-of-Principle Study for Acquired Brain Injuries
Source: J Clin Med. 2024 Jul 21;13(14):4254. doi: 10.3390/jcm13144254 (PMC11278066; doi:10.3390/jcm13144254)
Supplement: Supplementary file 1 [file jcm-13-04254-s001.zip › File S2 - ROSE Tool.pdf]

Rehabilitation Oculomotor Screening Evaluation (ROSE)

Date & Time:
Eye Pathology:
Double Vision: N / Y: Horizontal/Vertical

Glasses: N / Y: _____________
Corrected visual acuity (x/20):

Equipment Needed: 30cm ruler with a small circular sticker at each end, metronome (app), stopwatch (app), eye cover (optional), pen light, a paper with a size 12, Calibri, “E” in the centre.

Symptoms Rating in the last 24hrs: (Dizziness, Nausea, Headache, Fogginess, Eye pain, Other ….)

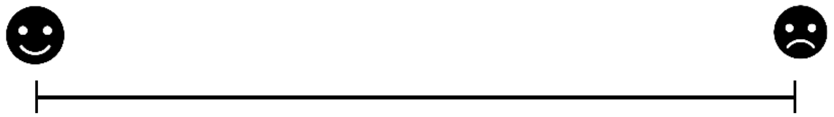

Observations<br>*Check all which apply*	
**All within normal limits**	☐
Small tilt/turn (<10 degrees) or small tremor	☐
Large tilt/turns (>10 degrees) or large tremor	☐
*Slow light reflex (PERRLA), or asymmetry	☐
*No light reflex (PERRLA)	(L) ☐ (R) ☐
Mild ptosis/retraction/lid fasciculation	(L) ☐ (R) ☐
Severe ptosis/retraction/lid fasciculation	(L) ☐ (R) ☐

Additional Notes:

Right Eye Left Eye

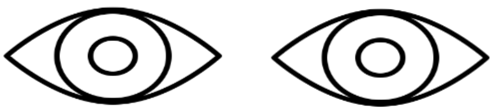

* This symbol is indicative of a symptom that would require follow up with specialized medical practitioner (general practitioner, optometrist, neurologist, ophthalmologist).

Smooth Pursuit

Hold a small fixation target 40cm from the subject's nasion. Instruct them to look at the target and follow it while it moves. The subject should hold their head still. If they are unable to comply, stabilize their head for testing. Slowly (<40°/s) move the target to a span of 45° from midline (~40cm). Perform 2 cycles in all three directions (there & back = 1 cycle). Look for deficits.

		Horizontal	Vertical
Movement Quality<br><br>*Small amplitude: ~2-5mm*<br>*Large amplitude: >5mm*	No jerkiness/ catch-up saccades **OR** less than 2 saccades	0	0
	Small amplitude **OR** 3-5 saccades	1	1
	Large amplitude **OR** >5 saccades	2	2
	Amplitude Subtotal	/4	

		Horizontal	Vertical	Vergence
Symmetry of eye movements	Symmetrical	0	0	0
	Minor asymmetry	1	1	1
	Major asymmetry	2	2	2
	# Of Saccades Subtotal	/6		
	**Smooth Pursuit Total Score**	**/10**		

Additional notes: Use the following figure to mark the location of the saccade(s) during smooth pursuits (i.e., Horizontal, Vertical or Vergence ⊗/⊙)

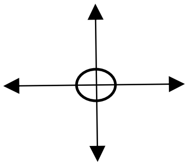

Vergence

Begin by holding a 30 cm ruler on the subject's mid forehead (2cm above nasion). Slowly move the target toward the subject's nasion. Record the distance when the subject reports seeing double or when one of the eyes shift outwards (whichever occurs first) (NPC). Slowly move back out and record the distance (cm) when subject reports seeing the target clearly (I.e., no longer double) (Recovery). Repeat 3x.
***If unable to test (no double vision), remove test from final score.*

Trial 1		Trial 2		Trial 3		Longest Distance	
NPC	Recovery	NPC	Recovery	NPC	Recovery	NPC	Recovery ∆
cm	cm	cm	cm	cm	cm	cm	cm

NPC Score	≤ 5.0cm of the nasion	0
	>5.0cm & <10cm of the nasion	1
	≥10cm of the nasion	2
Recovery ∆	≤ 7.0cm of the nasion	0
	>7.0 & <12 cm of the nasion	1
	≥12 cm of the nasion	2
	**Vergence Total Score**	**/4**

Symptom Rating (Smooth Pursuit + Vergence)

☺ ☹

Saccades

The subject will be looking back and forth between the 2 targets on the ruler that is 40cm away (15cm for vergence). Instruct the subject to look at the back and forth in between the two target as fast as possible for 8 seconds (using a timer). Count the number of cycles performed in each plane. Do not allow the subject to move their head. Look for signs of apraxia, hypometria, dysmetria, inaccuracy, ocular flutter, and overshoot.

	# of saccades:	Horizontal #: ___	Vertical #: ___	Vergence
Number of Saccadic Cycles *(there & back = 1 cycle)*	>7	0	0	0
	4-7	1	1	1
	<4	2	2	2
	# of Cycles Subtotal	/6		

		Horizontal	Vertical
Accuracy *Mild: Deviation easily corrected Severe: Consistent missing of target.*	Accurate	0	0
	Mild under/overshoot	1	1
	Severe under/overshoot	2	2
	Accuracy Subtotal	/4	

		Horizontal	Vertical	Vergence
Symmetry of mvts *Mild: Deviates yet synchronous Severe: deviates without synchrony*	Symmetrical	0	0	0
	Mild asymmetry	1	1	1
	Severe asymmetry	2	2	2
	Symmetry Subtotal	/6		
	**Saccades Total Score**	**/16**		

Additional notes:

Symptom Rating (Saccades)

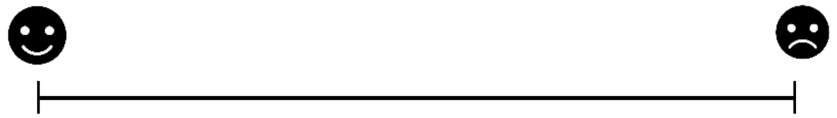

Fixation in 8 Gaze Directions

The subject is advised to focus on a stable target, such as the examiner's finger, positioned at the 45° (~40cm) in each gaze direction (vertical, horizontal, and diagonally) for at least 4 seconds (per direction). Ensure the subject's head is stabilized in a fixed position for testing. Can be performed immediately after smooth pursuits. Observe for gaze consistency, instability, loss of fixation, and nystagmus.

		Left	Right
Fixation	Stable fixation in 8 gaze directions. No signs of gaze induced nystagmus or drift. Able to maintain fixation in end of range gaze position for ≤ 4s.	0	0
	Gaze induced nystagmus and/or drift observed in 1 direction*.	1	1
	Gaze induced nystagmus and/or drift observed in >1 directions* or not end of range	2	2
	**Fixation Total Score**	**/4**	

Additional notes:

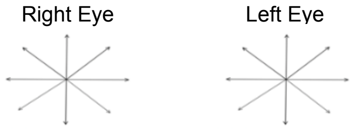

*This symbol is indicative of a symptom that would require follow up with specialized medical practitioner (general practitioner, optometrist, neurologist, ophthalmologist).

Eye Cover Test<br>*Ask the subject to keep looking at the examiner's nose. Cover one eye for 2s. Note if there is any corrective shift observed in the uncovered eye.*			
Eye Movement<br><br>*Score the eye that is* ***uncovered***		Left	Right
	Uncovered eye remains on target (i.e., no correction)	0	0
	Uncovered eye moves • outwards • inwards	1	1
	*Uncovered eye moves • upwards • downwards	2	2
	Subtotal Score	/4	
Alternate Cover-Uncover Test<br>*Ask the subject to look at the examiner's nose. Cover one eye for at least 1s before switching. Once an eye is occluded, the examiner observes the eye that was previously covered. Note the direction of any movement made on the image below. Perform at least 4x/eye.*			
Direction of Deviation		Left	Right
	Eyes remain on the target	0	0
	Uncovered eye moves mainly • outwards • inwards	1	1
	*Uncovered eye moves • upwards • downwards	2	2
	Subtotal Score	/4	
	**Total Eye Cover + Alternate Cover-Uncover Score**	**/8**	
Notes:			

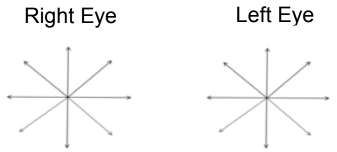

Symptom Rating (Cover Tests + Gaze fixation)

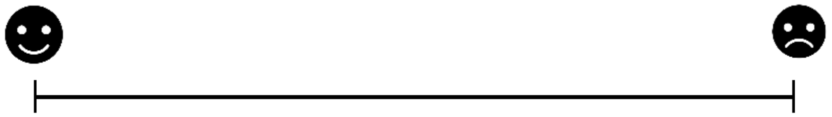

VOR Cancellation (cVOR)<br>*Ask the subject to sit with their feet on the floor, with their arms extended and hands clasped (thumbs up). While maintaining focus on the thumb, the examiner will rotate the subject's body as a whole, achieving a total of 80° rotation. Use a metronome set at 50bpm (1 beat/direction). Complete 5 cycles.*		
cVOR<br><br>*Mild: Deviation easily corrected*<br>*Severe: Consistent missing of target*	Eyes remain on the target	0
	Mild corrective saccades (under/overshoot)	1
	Severe corrective saccades (under/overshoot)	2
	Subtotal Score	/2

Vestibular ocular reflex (VOR)<br>*Ask the subject to fixate at a small target (Calibri, size 12, "E", at the centre of a cue card) placed on a table in front of them (20° of neck flexion). Move the subject's head from side to side (i.e., "no"), and then up and down (i.e., "yes") for 5 cycles, while maintaining fixation. Provide the cue "don't resist me," if the subject is tensing the neck. Move the head at 120bpm (1 beat/cycle), through a range of ±25-30°.*			
VOR		Horizontal	Vertical
	Stable target	0	0
	Blurry or jumping target	1	1
	Inability to see target (E)	2	2
	Subtotal Score	/ 4	
	**Total VOR + cVOR Score**	**/6**	

Symptom Rating (cVOR+VOR)

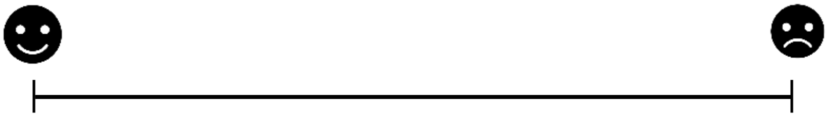

Test Item	Score	VAS	What is/are the most provocative
Baseline		/10	
Smooth Pursuits	/14	/10	
Saccades	/16	/10	
Cover Test + Gaze fixation	/12	/10	
VOR +cVOR Score	/6	/10	
**Total Score**	**/48**	**/50**	
**Total Score without Vergence**			

<u>Symptoms Rating Sheet</u>

1. Baseline: _________

None Severe

2. Smooth Pursuits & Vergence: _________

None Severe

3. Saccades: _________

None Severe

4. Cover Test & Gaze Fixation: _________

None Severe

5. cVOR & VOR: _________

None Severe
